# Construction and validation of the prediction model for fear of cancer recurrence in patients with postoperative cervical cancer

**DOI:** 10.3389/fonc.2025.1670680

**Published:** 2025-11-06

**Authors:** Chenyu Jia, Ningyan Li, Tingting Lu, Xintong Shen, Shuting Tang, Shoudi Hu, Guan Gui, Jinzhi Li

**Affiliations:** 1 College of Nursing, Bengbu Medical University, Bengbu, China; 2 Gynecologic Oncology Department, First Affiliated Hospital of Bengbu Medical University, Bengbu, China

**Keywords:** cervical cancer, fear of cancer recurrence, influencing factors, prediction model, nomogram

## Abstract

**Objectives:**

To construct a nomogram model for predicting the danger of fear of cancer recurrence in postoperative cervical cancer patients and to verify the predictive efficacy of the model.

**Methods:**

A total of 310 patients who underwent cervical cancer surgery at the Gynecologic Oncology Department of the First Affiliated Hospital of Bengbu Medical University from May 2024 to December 2024. The influencing factors were screened using single and multifactor stepwise logistic regression analysis. A nomogram prediction model was built using these predictors. Using 1,000 bootstrap resamples and the area under the curve(AUC) of the participants’ operating characteristics, the model’s effectiveness was confirmed.

**Results:**

Within the study population, 174 out of 310 patients(56.12%)exhibited a fear of cancer recurrence. Multifactorial analysis highlighted that variables such as age, educational level, treatment modality, Social Support Rate Scale(SSRS), and monthly family income significantly influenced fear of cancer recurrence in patients with postoperative cervical cancer(*P* < 0.05). Subsequently, a predictive model was established based on these factors. The model’s goodness-of-fit was assessed using the Hosmer-Lemeshow test, yielding a *χ*² value of 6.773(*P* = 0.610). The area under the receiver operating characteristic curve(AUC) was determined to be 0.910(95%CI 0.853-0.966), with a sensitivity of 87.5% and specificity of 81.2%.

**Conclusion:**

The research results indicate that the incidence of fear of cancer recurrence is high among them. Furthermore, the developed prediction model’s high predictive efficacy, suggesting its potential utility for individualized risk assessment concerning fear of cancer recurrence in this patient population. This model was developed and validated in a single-center cohort, and its generalizability requires future external validation.

## 1 Introduction

Cervical cancer is a critical global public health issue, profoundly impacting women’s health. According to the latest data provided by the International Agency for Research on Cancer (IARC), there were approximately 664,000 new cases and an estimated 35,000 deaths attributed to cervical cancer worldwide in 2024 (1). The National Cancer Centre of China’s National Cancer Report 2024 (2) indicates that China accounts for around 110,000 new cervical cancer cases annually, placing it seventh in the global incidence of female malignant tumors, with over 60,000 deaths each year. Despite the substantial enhancement in survival rates for early-stage patients due to aggressive surgery, the likelihood of recurrence remains. Clinical studies have confirmed that the recurrence rate of cervical cancer patients can be 10%-38% within 5 years after surgery (3–5). This persistent risk of recurrence places a significant psychological burden on survivors, most notably manifesting as fear of cancer recurrence(FCR). FCR represents a central psychological challenge in cancer survivorship with a high prevalence ranging from 37% to 84% (6–8). The defining features of FCR are characterized by persistent hypervigilance to bodily sensations, generalized anxiety, and social withdrawal. These symptoms collectively severely compromise patients’ quality of life, which impairs treatment adherence and elevates the risk of comorbid anxiety and depression. Research indicates that FCR arising from cognitive distortions or psychological avoidance behaviors can initiate a detrimental cycle: through pathways such as financial strain and social functioning limitations, it exacerbates mood disorders and increases the risk of psychiatric complications, thereby further reducing treatment adherence (9, 10). Recognized as a core issue in gynecological oncology rehabilitation by clinical guidelines, FCR necessitates systematic assessment and early intervention to mitigate its profound impact on long-term recovery. The Chinese Expert Consensus on Integrated Rehabilitation of Gynaecological Malignant Tumor (11) clearly states that FCR is the core psychological problem affecting the recovery of patients with cervical cancer, which needs to be systematically managed.

Researchers domestically and internationally have diligently investigated the development of FCR prediction models related to cancer. In 2003, Vickberg (12) identified the central role of FCR in the framework of cancer psychosocial responses. Previous research results (13–15) showed that younger age, married status, childbearing, depression and anxiety are independent risk factors for FCR in breast postoperative cancer patients, and the prediction weight of operation type and tumor stage for FCR in breast cancer patients is as high as 32.1%, and the probability of FCR in patients with stage III is 2.9 times that in patients with stage I. In response to the issue of increased risk of FCR in rural patients due to insufficient communication resources and inefficient social support utilization (16), Caumeil et al. (17) conducted a study on “integrating ecosystem barriers and promoting factors related to fear of cancer recurrence “, and found that symptom burden indirectly exacerbates FCR through the path of “loss of bodily control→inadequate ecological support”. A study (18) on the relationship between FCR, social support, and quality of life showed that individuals with lower FCR experienced higher levels of social support, leading to better quality of life. The consensus indicates that SSRS serves as a fundamental protective element, suggesting that elevated social support may alleviate patient concern by mitigating stress reactions and augmenting coping resources (19). The Chinese scholar Fang et al. (20) incorporated variables pertinent to cultural environment, hence enhancing the localized explanatory capacity of the FCR risk prediction model. Collectively, these investigations formulated a comprehensive prediction framework encompassing demographic attributes, disease and treatment variables, psychosocial factors and symptom burden. Notwithstanding the impressive outcomes, existing research continues to exhibit considerable deficiencies. Current models predominantly utilize cross-sectional data from a singular time point, neglecting the fluctuations of FCR in relation to disease duration. As research continues to deepen, evidence of the association between tumor markers (21), inflammatory factors (22), and FCR has emerged. However, existing prediction models rarely integrate such objective biological indicators. Current models focus on risk stratification, but how to drive personalized, stepped psychosocial interventions after risk stratification has not yet been developed as an accompanying strategy. There is a disconnect between model prediction and intervention implementation. Now, FCR has become a focus of clinical attention, but there are very few studies on the FCR in postoperative cervical cancer patients both domestically and internationally.

Despite the high prevalence and significant impact of FCR, routine postoperative care for cervical cancer largely emphasizes monitoring physiological parameters and managing physical complications. Unfortunately, there is a notable lack of systematic assessment and early intervention for psychological distress. The lack of structured and efficient screening tools often leads to FCR going undetected in busy clinical settings, escalating into severe anxiety or causing non-adherence to follow-up care. A nomogram (23) is a graphical calculating device that represents the mathematical model of the regression equation, allowing for an individualized visual prediction of the probability of FCR. The development and validation of FCR prediction models for postoperative cervical cancer patients in China is crucial for implementing targeted management of psychological risks and guiding early individualized interventions. This study aims to create a scientific prediction tool that provides an evidence-based foundation for enhancing patients’ long-term quality of life and optimizing nursing practices.

## 2 Methods

### 2.1 Participants

This study was employed a cross-sectional research design and conducted at the Gynecologic Oncology Department of the First Affiliated Hospital of Bengbu Medical University, a major tertiary care hospital and regional medical center in Anhui Province, China. As a leading institution in the region, it admits a substantial number of patients with gynecological malignancies from both urban and rural backgrounds, providing a study population that is broadly representative of cervical cancer patients in central China. We selected 310 postoperative cervical cancer patients who received treatment at this hospital from May to December, 2024. Eligible participants satisfied these criteria: 1) age≥18 years; 2) met the diagnostic criteria established by the National Comprehensive Cancer Network(NCCN) in the 2025 Clinical Practice Guidelines for Cervical Cancer (24) and were considered elective postoperative cases; 3) completed six months of follow-up, with comprehensive follow-up data and demonstrated a high adherence rate; 4)not experienced any major stressful events, such as bereavement, within the last six months; 5)in a good state of consciousness with no mental illness. The exclusion criteria were as follows: 1) individuals with a combination of infectious diseases, hematologic disorders, or significant cardiovascular, neurological, or pulmonary conditions; 2) with other medical conditions that may influence gynecologic tumor marker levels; 3) individuals with incomplete clinical case data. The event/variable method (25) was employed to determine the requisite sample size for the predictive model, necessitating a sample size of 10 to 20 times the number of predictors included in the study. In this investigation, a total of 22 predictors were identified, which indicated a required sample size of 220 to 440 cases. Ultimately, 310 postoperative cervical cancer patients were enrolled in the study. The participants were stratified into modeling and validation groups at a ratio of 7:3, resulting in 217 cases in the modeling group and 93 cases in the validation group. For participants who reported high levels of psychological distress during the study, a protocol was in place to provide information on available psychological support services and to facilitate referral upon the participant’s request. This study was reviewed and approved by the Ethics Committee of Bengbu Medical University in China (Lenko Grant No. [2023] 254). All participants in this study were informed of the research purpose and provided informed consent forms.

### 2.2 Measurements

Based on literature review (6, 26), clinical experience and data availability, this study included the following 22 influencing factors:

#### 2.2.1 General information questionnaire

Includes two parts: 1)general information of patients: age, education level, work status, residence status, monthly family income, marital status, BMI, FIGO stage, whether to give birth, whether to have a family history of cervical cancer, time from surgery to the present, whether there is any recurrence, time of reexamination, and treatment method (with or without radiotherapy and chemotherapy); 2)laboratory indicators: whether to be infected by HIV, tumor markers test, presence of lymphatic metastasis.

#### 2.2.2 Hospital anxiety and depression scale

It was compiled by Zigmond and Snaith RP (27) in 1983 and subsequently adapted for Chinese by Ye et al. (28) from Shanghai Medical University in 1993. The Chinese adaptation of the HADS scale comprises 14 items, featuring an anxiety subscale (HADS-A) and a depression subscale (HADS-D), with each item rated from 0 to 3, allowing for a total score range of 0 to 21 for both HADS-A and HADS-D. A total score of 21 indicates that a HADS score of 0 to 7 is classified as asymptomatic; a score of 8 to 10 suggests the possible presence of anxiety or depressive symptoms, while a score of 11 to 21 confirms the definite presence of such symptoms, with an overall Cronbach’s α coefficient of 0.80 for the scale. In 2024, Zhu et al. (29) examined 52 patients receiving radiotherapy for cervical cancer and assessed the Cronbach’s α coefficients for the depression scale in the Hospital Anxiety and Depression Scale(HADS), as well as the Cronbach’s α coefficient for the HADS-D scale. The Cronbach’s α coefficient for the depression scale of the HADS was 0.813, indicating strong reliability and appropriateness for Chinese people.

#### 2.2.3 Fear of progression questionnaire-short form

The unidimensional scale was created by Mehnert (30) in 2006 and was subsequently adapted by Wu et al. (31) into a two-dimensional framework encompassing physical health and social family, serving as a trustworthy and valid testing instrument in 2015. The Chinese version of the FoP-Q-SF has 12 items on a 5-point Likert scale, with response possibilities from ‘never’ to ‘always’, yielding scores from a minimum of 12 to a maximum of 60 points. A score of ≥34 signifies a clinically established level of concern of fear of cancer recurrence, with increasing values reflecting an increased amount of such dread. The internal consistency, as measured by Cronbach’s α, for the overall scale was 0.883, while the coefficients for the two dimensions were 0.829 and 0.812. In 2018, Cai et al. (32) examined 237 female breast cancer patients, revealing that the overall Cronbach’s α coefficients for the simplified scale of the Chinese version of the cancer patients’ fear of disease progression were 0.856, with the coefficients for the two dimensions being 0.838 and 0.842. The findings indicated that the scale had great reliability and was suitable for Chinese adults.

#### 2.2.4 Social support rating scale

Compiled by Xiao (33) in 1994, this work is based on international studies and demonstrated strong reliability and validity when applied to the cancer population. The Chinese edition of the SSRS comprises 10 items and 3 dimensions. Entries 6 and 7 utilize multiple choice scoring, awarding zero points for the response ‘no source’ and points for the response ‘the following sources’, which includes numerous sources. The remaining elements are evaluated separately, with the alternatives aligned to the scores sequentially. A total score of 66 indicates that an SSRS of 22 or lower signifies little social support, an SSRS between 23 and 44 indicates medium social support, and an SSRS between 45 and 66 denotes high social support. The overall Cronbach’s α coefficient for this scale was 0.858. In 2024, Guo et al. (34) examined 288 instances of leprosy patients, revealing that the Cronbach’s α coefficients for the overall scale and the subscales were 0.858, with the total score of the scale being 0. 0 and the total score of the subscales being 1.0. Cronbach’s α coefficients ranged from 0.50 to 0.89, indicating strong reliability for Chinese adults.

#### 2.2.5 Medical coping model questionnaire

Compiled by Feifel (35) in 1987, it was translated and amended by Shen et al. (36) in 2000 for clinical study on psychological stress in patients in China. The Chinese iteration of the MCMQ scale comprises 20 items over three dimensions: one confrontation dimension (items1, 2, 5, 10, 12, 15, 16, 19); two avoidance dimensions (items3, 7, 8, 9, 11, 14, 17); and one submission dimension (items4, 6, 14, 18, 20). A four-point Likert scale was employed, featuring options from ‘never’ to ‘frequently, ‘ with ascending scores. Entries 1, 4, 9, 10, 12, 13, 18, and 19 were reverse-scored, while the remaining entries received positive scores. One indicated complete noncompliance; two indicated less compliance; and three assigned a score of 3 for the submission dimension(items4, 6, 14, 18, and 20). A score of 2 indicates non-compliance; 3 signifies partial compliance; and 4 denotes full compliance. The Cronbach’s α coefficients for the confronting, evading, and conceding aspects were 0.69, 0.60, and 0.76, respectively. In 2020, Zheng et al. (37) examined 120 instances of mothers presenting at the clinic for postpartum pelvic floor dysfunction, revealing that the Cronbach’s α coefficients across the dimensions varied from 0.60 to 0.76. The Cronbach’s α coefficients for the three dimensions were calculated. The initial reliability scores were: Facing0.69, Avoiding0.60, and Yielding0.76; the retest reliability scores were: Facing0.64, Avoiding0.85, and Yielding0.67, demonstrating that the internal reliability and validity of the questionnaire are robust and suitable for Chinese adults.

#### 2.2.6 Nutrition risk screening rating scale(NRS2002)

The NRS-2002 scale, created by Kondrup et al. (38) in 2002 and subsequently amended by the Chinese Medical Association Section on Parenteral Enteral Nutrition (2008)serves as an illustrative example in 2008 for nutritional risk assessment in China. The scale comprises three principal modules including six evaluation items: one nutritional status module (BMI, recent weight loss, food intake); one illness severity module (disease diagnosis and metabolic stress); and one age-adjusted module (≥70 years of age, plus one point). A tiered grading system was employed, yielding a cumulative score of 7. Each indication received a score ranging from 0 to 3 based on the level of risk, with the cumulative score of the nutritional status and illness module indicating that a total score of ≥3 signifies the existence of nutritional risk. The aggregate Cronbach’s α coefficient of the scale was 0.886. Zhu et al. (39) performed nutritional screening for gastric cancer inpatients in 2025, revealing that the NRS2002 Cronbach’s α coefficient was 0.928, indicating strong reliability and applicability to Chinese adults.

### 2.3 Data collection methods

General information was obtained from the case room records, while a questionnaire was conducted with patients via telephone follow-ups in March and June post-surgery, upon completion of data collection, the two datasets were amalgamated, and the average mean difference was calculated. The researcher utilized plain language, eschewing any implications, and methodically articulated the questionnaire content for the respondents to complete on their behalf. Questionnaires with non-standard responses, identical alternatives, or incomplete answers were deemed illegitimate and eliminated from the study.

### 2.4 Statistical analysis

Predictor selection involved a two-step process: variables with *P* < 0.001 from univariate analyses were entered into a multivariable logistic regression model with stepwise selection. The data were analyzed utilizing SPSS version 27.0. For measurement data according to a normal distribution, findings were presented as means and standard deviations. Discrete data are summarized by counts and proportions. Comparative analyses utilized *χ*2 statistics or Fisher’s exact technique. Independent factors associated with FCR were identified using logistic regression in cervical cancer populations. The final model comprising predictors with *P* < 0.05, was used to construct a nomogram using R version 4.2.5. The model’s discrimination was evaluated by the Receiver Operating Characteristic (ROC) curve analysis in R, with an area under the curve (AUC)>0.9 considered outstanding. Model calibration was assessed using the Hosmer-Lemeshow test. Internal validation was performed via the Bootstrap method with 1,000 resamples in R to correct for overoptimism, yielding bias-corrected performance metrics including sensitivity, specificity, and accuracy. *P* < 0.05 was considered as statistically significant. The dataset was checked for completeness, and only cases with complete data for all analyzed variables were included in the model development.

## 3 Result

### 3.1 Occurrence of fear of cancer recurrence in postoperative cervical cancer patients

This study revealed that 174 postoperative cervical cancer patients with a total FoP-Q-SF score of ≥34 exhibited an overall incidence of 56.12%. In the modeling group, 134 out of 217 patients(61.75%) experienced FCR. In the validation group, 40 out of 93 patients (43.01%) exhibited similar FCR. The analysis revealed no statistically significant difference in the incidence of FCR between the modeling and validation groups (*χ*2 = 1.967, *P* = 0.134), and the baseline data were comparable, allowing for internal validation. Univariate analysis of factors influencing fear of cancer recurrence in postoperative cervical cancer patients (**Table 1**). 

**Table 1:** Univariate analysis of influencing factors of fear of cancer recurrence in postoperative cervical cancer patients (n=310).

Item	Non-FCR group(n=136)	FCR group(n=174)	*χ*^2^/*t*/*z*	*P*
Age(years)			*χ*2=28.15	<0.001
<40	12(8.8%)	48(27.6%)		
≥40	124(91.2%)	126(72.4%)		
Education level			*χ*2=16.35	<0.001
Junior high school and below	68(50.0%)	132(75.9%)		
High school/secondary	40(29.4%)	32(18.4%)		
College and above	28(20.6%)	10(5.7%)		
Residence status			*χ*2=3.210	0.201
Living alone	22(16.2%)	35(20.1%)		
Living with spouse and children	102(75.0%)	120(69.0%)		
Living with friends	12(8.8%)	19(10.9%)		
Work status			*χ*2=1.532	0.465
Unemployed	15(11.0%)	16(9.2%)		
Working	105(77.2%)	135(77.6%)		
Retired	16(11.8%)	23(13.2%)		
Marital status			*χ*2=58.62	<0.001
Married	125(91.9%)	85(48.9%)		
Unmarried/Divorced/Widowed	11(8.1%)	89(51.1%)		
Monthly family income			*χ*2=38.25	<0.001
<3000	8(5.9%)	68(39.1%)		
3000-6000	82(60.3%)	92(52.9%)		
6001-10000	32(23.5%)	12(6.9%)		
>10000	14(10.3%)	2(1.1%)		
Maternity			*χ*2=0.45	0.502
No	42(30.9%)	58(33.3%)		
Yes	94(69.1%)	116(66.7%)		
BMI			*χ*2=1.876	0.391
<18.5	12(8.8%)	15(8.6%)		
18.5-23.9	98(72.1%)	120(69.0%)		
≥24.0	26(19.1%)	39(22.4%)		
HPV infect			*χ*2=0.08	0.777
No	12(8.8%)	15(8.6%)		
Yes	124(91.2%)	159(91.4%)		
FIGO installments			*χ*2=3.48	0.176
Stage I	40(29.4%)	45(25.9%)		
Stage II + Stage III	90(66.2%)	120(69.0%)		
Stage IV	6(4.4%)	9(5.2%)		
lymphatic transfer			*χ*2=0.32	0.572
No	112(82.4%)	140(80.5%)		
Yes	24(17.6%)	34(19.5%)		
Treatment			*χ*2=34.82	<0.001
Surgery	75(55.1%)	35(20.1%)		
Surgery+radiotherapy	38(27.9%)	60(34.5%)		
Surgery+Chemoradiotherapy	23(16.9%)	79(45.4%)		
Recurrence			*χ*2=25.84	<0.001
No	125(91.9%)	110(63.2%)		
Yes	11(8.1%)	64(36.8%)		
Tumor Marker Tests	1.5±0.6	3.8±1.5	*t*=-14.37	<0.001
Anemia			*χ*2=1.327	0.249
No	118(86.8%)	145(83.3%)		
Yes	18(13.2%)	29(16.7%)		
Time since surgery			*χ*2=2.85	0.241
<6month	35(25.7%)	55(31.6%)		
6-18month	65(47.8%)	75(43.1%)		
>18month	36(26.5%)	44(25.3%)		
Postoperative complication			*χ*2=14.26	<0.001
No	122(89.7%)	125(71.8%)		
Yes	14(10.3%)	49(28.2%)		
Number of re-inspections			*χ*2=3.015	0.221
1 time	80(58.8%)	95(54.6%)		
2 times	40(29.4%)	60(34.5%)		
3 times	16(11.8%)	19(10.9%)		
HADS score	9.4±3.0	10.9±3.6	*t*=-1.842	0.066
SSRS score	45.8±7.7	32.1±8.3	*t*=-12.75	<0.001
MCMQ score	35.7±5.1	36.3±5.7	*t*=-0.672	0.502
NRS2002 score	2.2±0.7	3.9±1.3	*t*=-11.24	<0.001

### 3.2 Multifactorial analysis of the factors influencing the fear of cancer recurrence in postoperative cervical cancer patients

Those variables that achieved statistical significance in the univariate analysis of the influencing elements of fear of cancer recurrence for postoperative cervical cancer patients were utilized as independent variables, and whether there was FCR in postoperative patients with cervical cancer was set as a dependent variable for logistic regression analysis. The results of the multicollinearity assessment demonstrated that multicollinearity was not a problem. The results of the logistic regression showed that age(OR = 1.72, 95%CI 1.15-2.58), education level(OR = 2.01, 95%CI 1.32-3.07), treatment modality (OR = 1.89, 95%CI 1.24-2.89), Social Support Rating Scale(SSRS) score(OR = 0.67, 95%CI 0.51-0.88), and monthly family income(OR = 1.95, 95%CI 1.27-3.00) were influential factors in postoperative cervical cancer patients who has FCR(*P* < 0.05). The way of assigning values **Table 2** details predictor variables, while **Table 3** presents multivariate analysis results identifying determinants of postoperative FCR in cervical cancer patients.

**Table 2:** Independent variable assignment method.

Variable	Assignment method
Age	<40=1; ≥40=2
Education level	Junior high school and below=1;High school/middle school=2;College and above=3
Marital status	Married=1; Unmarried/Divorced/Widowed=2
Type of treatment	Surgery=1;Surgery+Radiotherapy=2; Surgery+Chemoradiotherapy=3
Monthly family income	<3000=1;3000-6000=2;6001-10000=3;>10000=4
Recurrence	No=1;Yes=2
Postoperative Complications	No=1;Yes=2
Tumor Markers	Original value substitution
SSRS score	Substitution of original values
NRS2002 score	Substitution of original values

**Table 3:** Multivariate logistic regression analysis of influencing factors for fear of cancer recurrence in postoperative cervical cancer patients.

Variable	B	SE	Wald	*P*	OR	95%CI
Constant	-1.572	0.402	15.284	<0.001	–	–
Age	0.544	0.140	15.11	<0.001	1.72	[1.15-2.58]
Education level	0.698	0.142	24.15	<0.001	2.01	[1.32-3.07]
Marital status	0.310	0.310	1.00	0.317	1.36	[0.74-2.50]
Treatment	0.637	0.143	19.86	<0.001	1.89	[1.24-2.89]
Relapse	0.350	0.350	1.00	0.317	1.42	[0.72-2.82]
Monthly Family Income	0.668	0.144	21.53	<0.001	1.95	[1.27-3.00]
Postoperative Complications	0.982	0.32	9.42	0.002	2.67	[1.43-5.00]
Tumor Markers	0.140	0.110	1.62	0.203	1.15	[0.93-1.42]
SSRS score	-0.401	0.096	17.45	<0.001	0.67	[0.51-0.88]
NRS2002 score	0.070	0.130	0.29	0.591	1.07	[0.83-1.38]

### 3.3 Construction of FCR prediction model for postoperative cervical cancer patients

**Figure 1:**
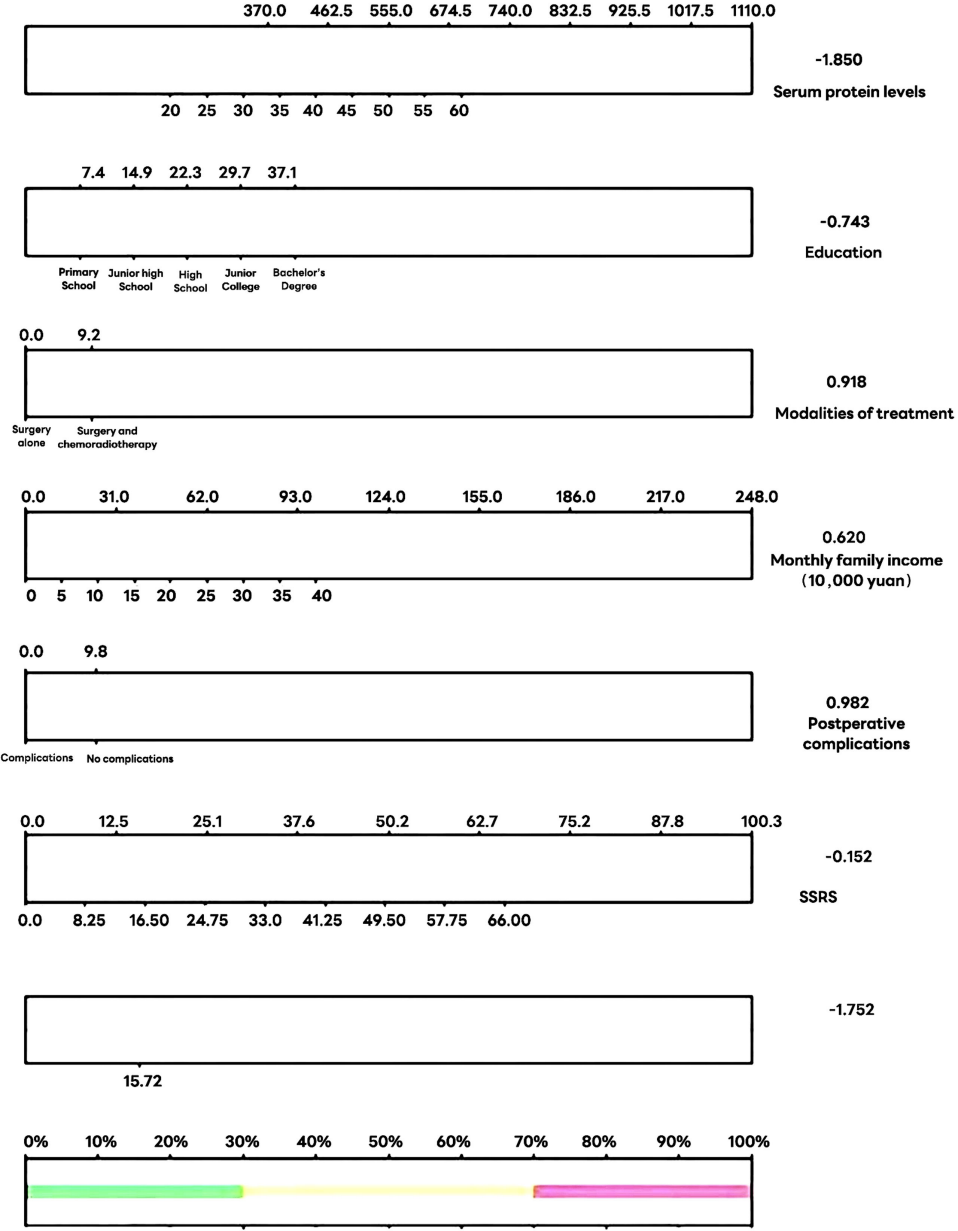
Column line plot of predicted risk of fear of cancer recurrence in postoperative patients with cervical cancer.

The FCR prediction model for postoperative cervical cancer patients was constructed according to the factors influencing the FCR postoperative cervical cancer patients, Logit(*P*)=-1.572-1.850 serum albumin level-0.743×education level+0.918×treatment modality-0.620×monthly family income+0.982×postoperative complications-0.152 ×SSRS. A line graph is shown in **Figure 1**.

### 3.4 Results of the goodness-of-fit test for the model predicting the fear of cancer recurrence in postoperative cervical cancer patients

**Figure 2:**
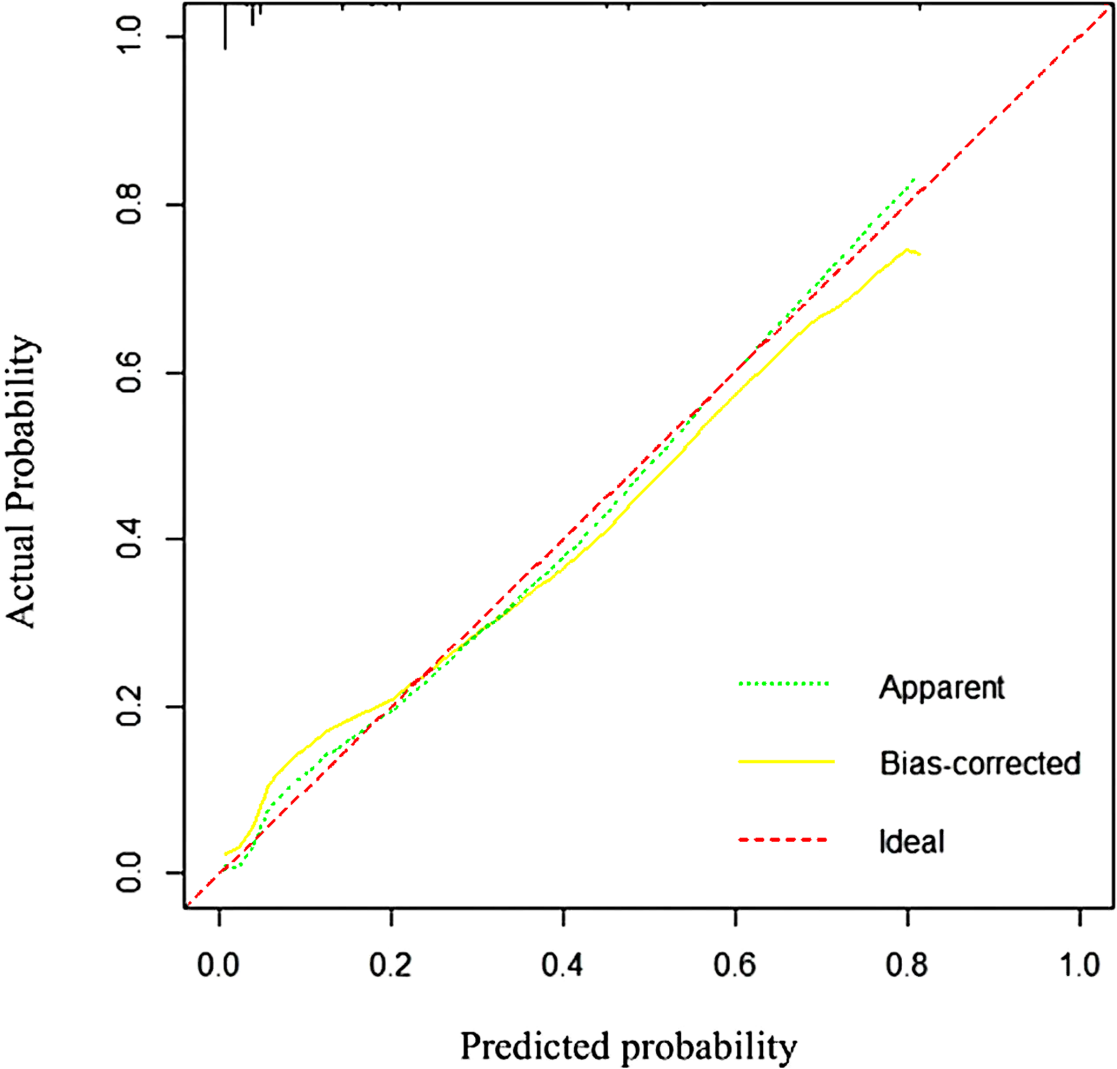
Subject operating characteristic curves for the fear of cancer recurrence risk prediction model in postoperative cervical cancer patients.

ROC curves, Hosmer⁃Lemeshow tests and calibration plots were accustomed to assess the performance of the model. Significant association was observed(χ²=6.773). The ROC curve achieved 0.910 AUC(95%CI 0.853-0.966), demonstrating 87.5% diagnostic sensitivity and 81.2% specificity (**Figure 2**). It indicates excellent model discrimination. According to the grading guidelines, this indicates that the model in this study has excellent discrimination. The Hosmer-Lemeshow test yielded *P* = 0.610, demonstrating adequate model calibration with no statistically significant deviation between predicted and observed outcomes.

### 3.5 Validation results of the prediction model for the risk of fear of cancer recurrence in postoperative cervical cancer patients

**Figure 3:**
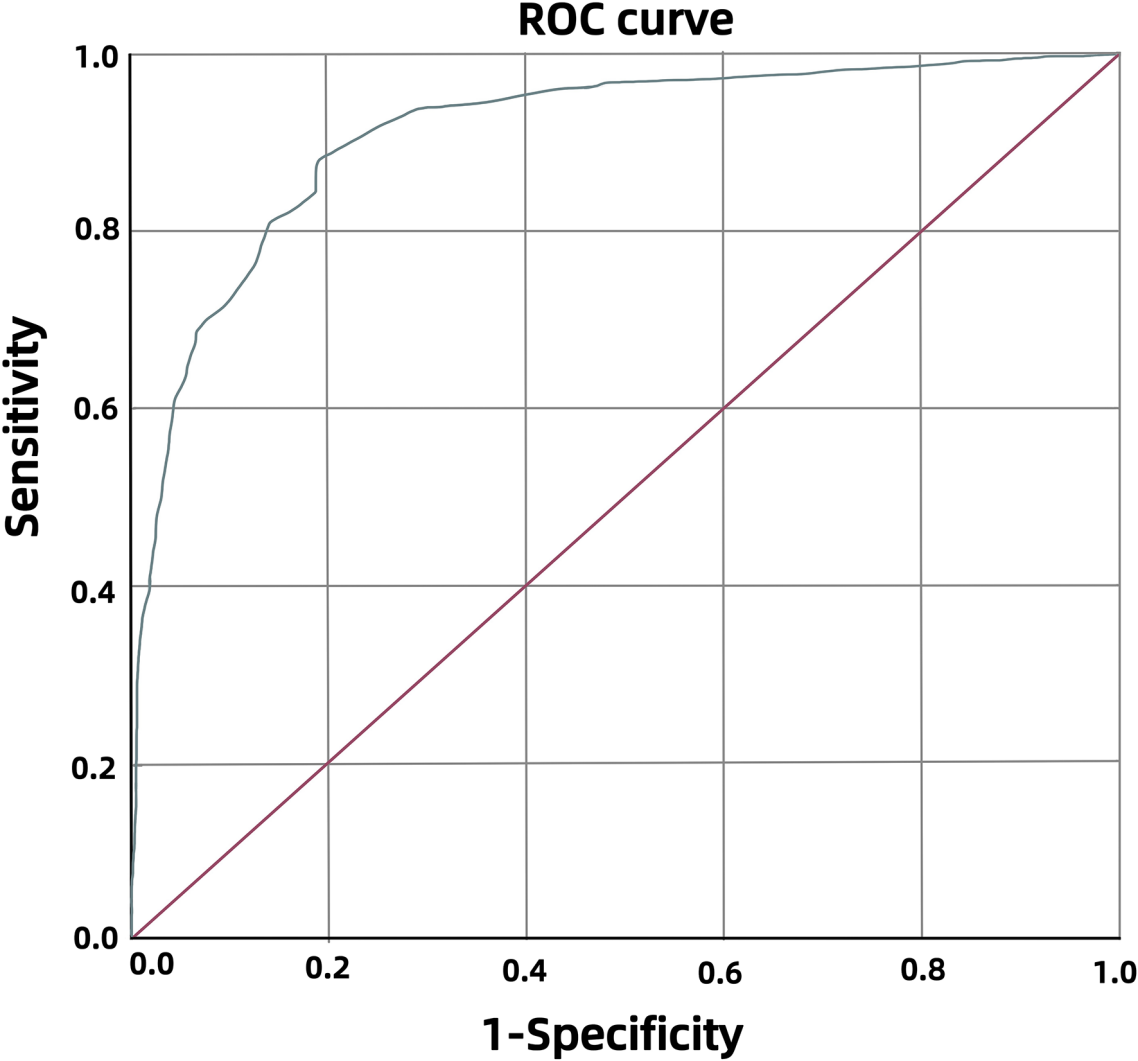
Calibration curves predicted by the risk prediction model.

The model calibration is plotted (**Figure 3**), and the prediction curve is basically consistent with the ideal curve, with an average absolute error of 0.02. The Bootstrap self-help method is used to resample the data 1, 000 times to validate this model underwent internal validation, yielding a concordance index (C-index) of the bias-corrected Summers Dxy rank correlation, and R-squared exponent (R2) in the original set are 0.910, 0.820, and 0.505; in the test set, they are 0.909, 0.819 and 0.481, indicating that the predictions using the original and test sets are consistent.

## 4 Discussion

### 4.1 Higher incidence of FCR in postoperative patients with cervical cancer

The results of this study showed that the incidence of FCR in postoperative cervical cancer patients was 56.12%, which was similar to the incidence rate of 57.02% reported by Zhou et al. (40), and higher than the incidence rate of 49% of FCR in general cancer patients (41). Numerous investigations on individuals with solid tumors have demonstrated significant variability in the prevalence of fear of cancer recurrence. In contrast to other malignancies, the proximity of cervical cancer foci to lymph nodes (42), blood vessels, and adjacent organs is less conspicuous. Cervical cancer affects reproductive fertility (43)and is associated with sexually transmitted beliefs, which contribute to a sense of shame. This, in turn, heightens the psychological burden and exacerbates the fear of cancer recurrence. A survey indicated that the prevalence of fear of cancer recurrence among breast cancer patients was approximately 52.9% (44), while another study revealed that the frequency of fear of cancer recurrence during neoadjuvant chemotherapy in cervical cancer patients was 66.7% (45). The disparities may be attributed to the current study’s emphasis on the postoperative phase, during which patients endure the compounded stress of recovering from treatment trauma and grappling with long-term prognostic uncertainty, resulting in heightened psychological vulnerability. The Chinese version of the FCR Scale used in this study has high sensitivity and may have identified more subclinical manifestations of fear responses.

### 4.2 Factors influencing the occurrence of fear of cancer recurrence in postoperative patients with cervical cancer

This study’s multifactorial logistic regression analysis identified age (OR = 1.72, 95%CI 1.15-2.58), education level (OR = 2.01, 95%CI 1.32-3.07), treatment modality (OR = 1.89, 95%CI 1.24-2.89), Social Support Rating Scale (SSRS) score (OR = 0.67, 95%CI 0.51-0.88), and monthly family income (OR = 1.95, 95%CI 1.27-3.00) as independent risk factors for fear of cancer recurrence of postoperative recurrence in cervical cancer patients.

The probability of fear of cancer recurrence was greater in younger patients compared to older patients, aligning with the findings of Hu et al. (46). Younger women are at a pivotal juncture in their professional development and family planning, coupled with a longer life expectancy, which amplifies their apprehension regarding disease recurrence; concurrently, they frequently bear the responsibility of child-rearing, leading to heightened vigilance concerning the progression of their illness. Nevertheless, owing to their limited life experience, they possess diminished psychological resilience to manage the significant upheaval of cancer and exhibit an elevated fear of cancer recurrence. Compared to younger patients, elderly patients have a stronger tolerance for disease recurrence, which is attributed to their extensive life experience. Therefore, healthcare professionals should give special consideration to personalized treatment plans for young cancer patients, harmonize their physical recovery and psychological support in nursing care, provide guidance on healthy lifestyles while connecting resources to mitigate, thereby helping patients regain confidence and reducing fear of disease recurrence.

Individuals with limited educational attainment are susceptible to fear of cancer recurrence. A cross-sectional survey of 100 patients undergoing cervical cancer radical surgery showed that patients with lower education levels had poor cognitive abilities and weak understanding of the disease, which led to problems such as lack of understanding of the disease, information blockage, disagreement with the treatment plan, or excessive concern about the disease during the treatment process, increasing their risk of fear of cancer recurrence (47). The underlying mechanism may involve health literacy and coping strategies. Lower education often correlates with decreased ability to access, understand, and appraise complex medical information, leading to misconceptions about prognosis and recurrence signals. This knowledge gap can foster maladaptive coping mechanisms, such as catastrophizing or avoidance, thereby amplifying FCR. Healthcare professionals should elucidate disease knowledge and treatment plans using accessible language, visuals, and other intuitive methods for patients with low education; conduct regular follow-ups and proactively address inquiries and concerns; and guide family members in facilitating information dissemination to dismantle informational barriers and mitigate patients’ cognitive obstacles and anxieties.

Related studies (48, 49) have shown that cervical cancer patients treated with radiotherapy and chemotherapy have an increasing fear of disease progression as treatment time progresses, indicating that such patients are more likely to experience fear of cancer recurrence. The unique anatomical positioning of cervical cancer, coupled with its biological propensity for lymph node metastasis, complicates the complete eradication of cancer cells through intracavitary radiotherapy. Additionally, the substantial side effects related to treatment, including fatigue, nausea, pain, and skin reactions, as well as prolonged treatment duration (50) and considerable physical and psychological strain, will reduce patients’ confidence in treatment and significantly increase fear of cancer recurrence, with a significantly increased risk of FCR. Healthcare practitioners must closely monitor these patients, recognize early indicators of heightened relapse fear, and create focused intervention programs to mitigate the dread of illness progression.

A low monthly family income correlates with an increased likelihood of fear of cancer recurrence in postoperative cervical cancer patients, aligning with findings from other studies on colorectal cancer patients (51). This association may be explained by the financial toxicity of cancer treatment. Patients with limited economic resources face heightened anxiety about the costs of long-term surveillance, potential recurrence treatments, and loss of income due to disability, which can conflate general survival anxieties with specific fears of recurrence. It may arise from their various apprehensions of long-term survival, familial obligations, and reproductive issues. A study (52) involving 206 patients who underwent radical cancer surgery demonstrated that although healthcare insurance reform and other measures greatly reduced the direct treatment costs for patients, the prolonged duration of postoperative chemotherapy and radiotherapy can seriously damage patients’ normal work ability and quality of life, leading to a significant decrease or even interruption in their income. The decrease in income resulting from therapy might substantially elevate the financial strain and anxiety around disease recurrence for individuals with limited family resources. Therefore, healthcare providers can alleviate patients’ financial burdens and reduce FCR by educating family caregivers on disease knowledge systems, establishing mutual support groups, offering financial counseling and psychosocial education, and flexibly adjusting care plans.

Postoperative cervical cancer patients exhibiting elevated social support experience a diminished incidence of FCR. This protective effect can be attributed to the stress-buffering model of social support (53). Strong social networks provide tangible assistance (e.g., accompanying to appointments), emotional comfort, and informational guidance, which enhance patients’ perceived control and adaptive coping capacities. This support system helps reframe threats and reduces the sense of isolation that often exacerbates FCR. This study utilized the SSRS scale score to assess patients’ social support. Patients experiencing low social support became disengaged from work and social interactions due to cancer treatment, yearning for the care of family and friends. However, they faced alienation from social groups (54) as a result of cervical cancer and indifference from family members (55)which heightened the risk of FCR. Nursing personnel ought to guide family members to provide them care and acceptance, facilitate patients in reconstructing their social networks, promote involvement in patient support groups, so that help them reintegration into the workforce and daily life, and bolster psychological counseling to mitigate the fear of cancer recurrence stemming from feelings of alienation. Hence, structured counselling programs and peer-led educational initiatives should be developed to strengthen perceived support, improve coping skills, and directly address fears of recurrence in this vulnerable subgroup. The interplay of these intricate elements elucidates the significant incidence of FCR and identifies a specific focus for future intervention.

### 4.3 Implications for interventions

This model facilitates a stepped-care approach to psychosocial support. For patients stratified as high-risk, our findings recommend immediate referral for evidence-based interventions such as cognitive-behavioral therapy (CBT) (56) tailored for FCR or mindfulness-based stress reduction (MBSR) (57), which have proven efficacy in mitigating cancer-related distress. For low-risk patients, standardized education on recurrence signs and supportive coping strategies may suffice. This risk-stratified framework ensures that intensive resources are allocated efficiently while still providing a baseline of support to all patients, thereby enhancing the model’s translational impact.

### 4.4 A risk prediction model for fear of cancer recurrence in patients with postoperative cervical cancer is really beneficial

This study conducted a thorough review of 310 postoperative cervical cancer patients and showed that the overall incidence of fear of cancer recurrence among postoperative patients was 56.12%, which significantly surpasses the documented rate of FCR in patients with postoperative cervical cancer in prior literature (41), indicating that the population with cervical cancer has obvious psychological crisis characteristics. The elevated incidence rate signifies the widespread occurrence of postoperative psychological trauma among cervical cancer patients. In clinical practice, the postoperative treatment phase should represent an optimal period for physiological recovery; however, over fifty percent of patients remain persistently affected by the fear of cancer recurrence. This fear not only diminishes treatment adherence and quality of life but may also trigger the release of inflammatory mediators via the activation of the hypothalamo-pituitary-adrenal axis (58), thereby establishing a detrimental cycle from psychological anxiety to physiological symptoms.

The validated nomogram demonstrated excellent discriminative ability (AUC = 0.910, 95% CI: 0.853–0.966) and good calibration (Hosmer–Lemeshow test, P = 0.610). According to common interpretive guidelines (59), an AUC above 0.9 is considered ‘outstanding’ or ‘excellent’. This implies that our model has a high probability (91.0%) of correctly ranking a patient with FCR as higher risk than a patient without FCR. With a sensitivity of 87.5% and specificity of 81.2%, the model effectively identifies high-risk patients while accurately targeting those needing intensive support. Unlike the 25-minute FCR Scale (27), this tool uses only five routine clinical variables, making it highly suitable for rapid triage in Chinese outpatient settings. This model quantifies risk categorization, providing a pragmatic screening tool for integration into routine follow-up to flag high-risk patients needing proactive psychological support. Therefore, moves beyond mere risk prediction to offer a concrete strategy for guiding personalized, timely psychosocial support, addressing a critical gap in current postoperative care for cervical cancer survivors.

### 4.5 Limitations

As it exclusively involved patients with cervical cancer at 6 months postoperatively, Therefore, only immediate complications are included and not yet long-term complications such as neurogenic bladder dysfunction commonly occurs in the distant future after radical surgery (60), sexual dysfunction (61), and chronic lymphedema following extensive lymph node dissection (62) after radical surgery. Not only do these complications continue to impair quality of life, but they also lead to a subconscious interpretation of such persistent problems that the cancer is not truly cured, reinforcing the fear of cancer recurrence. Furthermore, the sample originated from a singular source, and external validation of the prediction model was not conducted, multicenter external validation studies should be conducted. A longitudinal study with a large sample size and multiple follow-up assessments should be conducted to track the dynamic evolution of FCR. Researchers should comprehensively collect various influencing factors of FCR in order to further validate and refine the model, ensuring its broader applicability and effectiveness in predicting postoperative fear of cancer recurrence in patients with cervical cancer.

## 5 Conclusion

This study introduces a practical prediction model for fear of cancer recurrence (FCR) in postoperative cervical cancer patients. By integrating five key predictors into a user-friendly nomogram, the model allows for the early identification of patients who are at high risk for FCR. We recommend implementing this tool in clinical practice to guide a stepped-care approach: providing cost-effective education to low-risk patients while ensuring early psychological intervention for those at high risk. This strategy aims to optimize supportive care resources and directly address the significant psychological burden experienced by survivors, ultimately leading to improved long-term patient outcomes.

## Data Availability

The raw data supporting the conclusions of this article will be made available by the authors, without undue reservation.
